# Untargeted metabolomic analysis uncovers metabolic variability of four *Bifidobacterial* strains for probiotic development

**DOI:** 10.3389/fmicb.2025.1522036

**Published:** 2025-01-31

**Authors:** Kailong Liu, Guoqiang Yao, Zhan Yang, Tian Huang, Lai-Yu Kwok, Heping Zhang

**Affiliations:** ^1^Key Laboratory of Dairy Biotechnology and Engineering, Ministry of Education, Inner Mongolia Agricultural University, Hohhot, China; ^2^Key Laboratory of Dairy Products Processing, Ministry of Agriculture and Rural Affairs, Inner Mongolia Agricultural University, Hohhot, China; ^3^Inner Mongolia Key Laboratory of Dairy Biotechnology and Engineering, Inner Mongolia Agricultural University, Hohhot, China; ^4^Collaborative Innovative Center for Lactic Acid Bacteria and Fermented Dairy Products, Ministry of Education, Inner Mongolia Agricultural University, Hohhot, China

**Keywords:** *Bifidobacterium animalis* subsp. *lactis*, *Bifidobacterium longum* subsp. *infantis*, *Bifidobacterium breve*, bioactive compounds, untargeted metabolomic

## Abstract

*Bifidobacterium* species are essential members of the human gut microbiota, playing crucial roles in host health. Variations in the metabolic functions of different *Bifidobacterium* strains can have distinct health effects, making it essential to understand their metabolic characteristics for the development of targeted probiotic formulations. In this study, we cultivated four selected *Bifidobacterium* strains from three species: *Bifidobacterium animalis* subsp. *lactis* BB-69, Bbm-19, *Bifidobacterium brevis* BX-18, and *Bifidobacterium longum* subsp. *infantis* B8762. We conducted an untargeted comparative metabolomic analysis to investigate the intracellular metabolic profile and functional activities of these strains. A total of 1,340 metabolites were identified, revealing significant variations in their metabolomic profiles. Notably, *Bifidobacterium animalis* subsp. *lactis* Bbm-19 showed higher activity in amino acid biosynthesis, while *Bifidobacterium animalis* subsp. *lactis* BB-69 exhibited increased activity in secondary bile acid biosynthesis and alpha-linolenic acid metabolism. *Bifidobacterium longum* subsp. *infantis* B8762 demonstrated enhanced activity in polycyclic aromatic hydrocarbon degradation, vitamin digestion and absorption, and galactose metabolism. *Bifidobacterium breve* BX-18 was more active in tryptophan metabolism and pentose and glucuronate interconversions. Targeted analyses of specific metabolites further revealed strain-specific metabolic pathways. This study systematically elucidates the metabolic profile differences among the four investigated *Bifidobacterium* strains at the untargeted metabolomic level, providing valuable insights into their metabolic characteristics and potential applications in probiotic development.

## 1 Introduction

Probiotics are “live microorganisms which when administered in adequate amounts confer a health benefit on the host” (Pereira et al., 2024). They offer a diverse range of health benefits primarily by maintaining gut homeostasis (Chen et al., 2023) and enhancing immunity (Kurian et al., 2021). They are utilized not only in the food and pharmaceutical industries but also widely implemented for disease prevention and treatment (O’Morain et al., 2020). Recent studies have highlighted that non-viable components and metabolites of probiotics can also promote health (Li et al., 2024). Probiotics or their metabolites have the properties of regulating immunity, alleviating allergies, regulating gastrointestinal function, antioxidant, anti-inflammatory (Li et al., 2020), as well as lowering blood sugar, preventing dental caries, anti-tumor, lowering blood pressure, etc., such as irritable bowel syndrome, allergic diseases and periodontitis. The active products synthesized by probiotics have the advantages of safety, stability, and easy storage and transportation, and have broad application prospects in the fields of food and medicine and health. Among various common probiotic taxa, *Bifidobacterium* species are crucial members of the human gut microbiota, including *Bifidobacterium longum* subsp. *infantis*, *Bifidobacterium bifidum*, *Bifidobacterium breve*, *Bifidobacterium animalis* subsp. *lactis*, and *Bifidobacterium adolescentis* (Bezkorovainy, 2020). *Bifidobacterium* exhibit numerous probiotic effects, such as improving intestinal disorders associated with enteritis (Khailova et al., 2009), enhancing the host’s antioxidant capacity (Kim et al., 2020), and alleviating constipation (Zhao et al., 2023). Additionally, some of these probiotics can regulate the nervous system by synthesizing neuroactive metabolites and upregulating acetylcholine levels, thereby enhancing neurological excitability (Wang et al., 2016). Currently, various bifidobacterial strains are utilized in the formulation of infant formula, functional fermented foods, and other health-promoting products. Therefore, research into their biological activities is crucial for enhancing these applications and maximizing their health benefits.

Metabolomics involves the qualitative and quantitative analysis of low-molecular-weight molecules using high-throughput techniques to capture changes in small molecules, providing insights into biological processes and nutrition-related metabolic pathways (Creydt et al., 2022). Untargeted metabolomics has recently gained prominence in various fields, including crop improvement (Razzaq et al., 2019), quality control of natural products (Creydt et al., 2018), and biomarker development (Cui et al., 2019). For example, a previous study applied targeted metabolomics based on ultra-high performance liquid chromatography coupled with Fourier transform mass spectrometry and untargeted metabolomics based on gas chromatography-mass spectrometry to predict the gene expression patterns in tomatoes (Tohge et al., 2020). Another study investigated the dynamic metabolic changes in anthocyanin accumulation during the color transformation process of Sichuan pepper fruit peel to enhance its visual appeal and quality (Wang et al., 2022). However, reports on the differences in bioactive metabolic profile among probiotic bacterial strains remain limited.

In 2015, the team completed the genome determination of 213 strains of *Lactobacillus* model strains, and found the nutrient metabolism rules of lactose metabolism and proteolysis (Sun et al., 2015). In addition, our team established a non-targeted metabolomics based on a rapid and effective detection method for the screening of functional probiotics at the strain level, and found that there was a huge difference in the intracellular metabolic profile between the two strains of *Bifidobacterium animalis* subsp. *lactis* (Wang et al., 2021).

This study focuses on four specific *Bifidobacterium* strains: *Bifidobacterium animalis* subsp. *lactis* BB-69, *Bifidobacterium animalis* subsp. *lactis* Bbm-19, *Bifidobacterium breve* BX-18, and *Bifidobacterium longum* subsp. *infantis* B8762. Using liquid chromatography-quadrupole time-of-flight mass spectrometry, we conducted a metabolomic analysis to investigate the differential metabolic profiles of these strains. Our aim was to provide a technical foundation for analyzing the functional characteristics of representative bifidobacterial components in probiotic formulations. The knowledge generated in this study will offer valuable insights into basic understanding of the physiology of different bifidobacterial strains and enhance their potential applications in functional product development.

## 2 Materials and methods

### 2.1 Microbial strains

In this study, four bifidobacterial strains were investigated: BB-69, Bbm-19, BX-18, and B8762. The BB-69 and B8762 strains were both isolated from the intestinal tract of healthy infants in 2017, while the BX-18 strain was isolated in 2018 from the feces of a long-lived elderly person in Enshi, Hubei. In contrast, the Bbm-19 strain was isolated from healthy breast milk in 2017. All strains were preserved and obtained from the Key Laboratory of Dairy Biotechnology and Engineering, Ministry of Education, Inner Mongolia Agricultural University, China. The complete genome sequences of the four *Bifidobacterium* strains have been deposited in the iLABdb database,^1^ a specialized database for lactic acid bacteria genomes (Jin et al., 2023). The genomic data, including genome assembly and annotation, can be accessed through the website or by email request. These strains are also available to researchers through formal material transfer agreement procedures.

### 2.2 Simulated gastrointestinal fluid tolerance test

In the modified MRS liquid medium sterilized at pH 2.5 (adjusted with 1 mol/L HCl), 3.5 g/L pepsin was added, and the sterilization was filtered and sterilized with a 0.22 μm microporous filter membrane to make a simulated gastric juice.

In the modified MRS liquid medium sterilized at pH 8.0 (adjusted with 0.1 mol/L NaOH), 0.10% trypsin and 1.80% bovine bile salt were added and sterilized by 0.22 μm microporous filter membrane to prepare a simulated intestinal fluid.

We added 5 × 10^7^ CFU/mL of the prepared bacterial solution to the simulated gastric juice, incubated at 37°C for 3 h, and measured the number of viable bacteria at 0 h and 3 h by the modified MRS agar medium pouring method. The bacterial artificial gastric juice with a pH of 2.5 was taken and cultured for 3 h, transferred into the artificial intestinal fluid, and the number of viable bacteria was measured by sampling after 8 h of anaerobic culture at 37°C, all anaerobic operations are carried out at the anaerobic workstation and the survival rate was calculated as follows.


$$\mathrm{Survival rate}=\left[N_{1}/N_{0}\right]\times 100\%$$

$$\mathrm{Survival rate}=\left[N_{2}/N_{1}\right]\times 100\%$$

*N*_0_—0 h number of viable bacteria; *N*_1_—the number of viable bacteria after 3 h of digestion by simulated gastric juice; *N*_2_—Number of viable bacteria after 8 h digestion of simulated intestinal fluid.

### 2.3 Chemicals and culture media

High-performance liquid chromatography-grade methanol and acetonitrile (Merck, Germany) and formic acid (Shanghai Aladdin Bio-Chem Technology Co., Ltd., Shanghai, China) were used in the study. The modified MRS liquid medium comprised the following components: (10 g/L), beef extract (5 g/L), yeast extract (4 g/L), glucose (20 g/L), Tween-80 (1.08 g/L), potassium dihydrogen phosphate (2 g/L), sodium acetate (5 g/L), ammonium citrate tribasic (2 g/L), magnesium sulfate heptahydrate (0.2 g/L), manganese sulfate tetrahydrate (0.05 g/L), and L-cysteine hydrochloride (0.5 g/L). The final pH of the medium was adjusted to 6.20 ± 0.02 using 1 mol/L hydrochloric acid or 1 mol/L sodium hydroxide.

### 2.4 Strain reactivation and growth

The test strains were obtained as frozen stock stored at −80°C. They were inoculated and reactivated in the modified MRS liquid medium, grown under constant temperature at 37°C. Following reactivation, the test strains were continued to grow as seed culture (2% cell density) at 37°C for 18 h. Subsequently, they were inoculated into fresh MRS liquid medium (5% cell density) for further cultivation at 37°C. Constant temperature anaerobic culture at 37°C to stable period. All strains were maintained under anaerobic conditions throughout the entire cultivation period. Microbial morphology was observed during the passage to ensure the purity of the test strains.

### 2.5 Preparation of cell metabolites

All strains were cultured until the end of the logarithmic phase, at which point the cultivation was terminated. They were then sterilized and inactivated at 90°C for 15 min. Following this, the inactivated broth was centrifuged at 14,000 rpm to pellet the cells, which were washed three times with sterile purified water and then subjected to vacuum freeze-drying. The samples were stored at −80°C until extraction of intracellular metabolites.

### 2.6 Extraction of intracellular metabolites

The frozen samples were thawed on ice. Approximately 40 ± 1 mg of each sample was weighed into a centrifuge tube, homogenized using a ball mill, and then centrifuged at 3,000 rpm for 30–50 s at 4°C. Then, 400 μL of 80% methanol–water internal standard extraction agent was added, and the mixture was vortexed for 3 min to ensure complete suspension of the sample. The sample was rapidly frozen in liquid nitrogen for 5 min, thawed on ice, vortexed for another 3 min, and this process was repeated three times. After centrifugation at 12,000 rpm for 10 min at 4°C, 300 μL of the supernatant was transferred to a new centrifuge tube. The extraction process was repeated under the same conditions to obtain an additional 200 μL of supernatant for analysis. Samples were filtered and transferred to appropriate sample vials for subsequent metabolomics analysis (Grün et al., 2019).

### 2.7 Liquid chromatography-quadrupole time-of-flight mass spectrometry

The analysis was performed on an ultra-performance liquid chromatography (UPLC) (ExionLC AD; AB Sciex LLC, Framingham, MA, United States) and tandem mass spectrometry (MS/MS) (QTRAP; AB Sciex LLC, Framingham, MA, United States).

#### 2.7.1 Chromatography conditions

The chromatography column used was a Waters ACQUITY UPLC HSS T3 C18 (1.8 μm, 2.1 mm × 100 mm). The mobile phase comprised Phase A (ultrapure water with 0.1% formic acid) and Phase B (acetonitrile with 0.1% formic acid). The elution gradient was as follows: at 0 min, water/acetonitrile (95:5 v/v); at 2.0 min (80:20 v/v); at 5.0 min (40:60 v/v); at 6.0 min (1:99 v/v); at 7.5 min (1:99 v/v); at 7.6 min (95:5 v/v); at 10.0 min (95:5 v/v). The flow rate was set to 0.4 mL/min, and the column temperature was maintained at 40°C. An injection volume of 2 μL was utilized (Chen et al., 2013).

#### 2.7.2 Mass spectrometry conditions

The electrospray ionization source temperature was set at 500°C, with a mass spectrometry voltage 5,500 V (positive) and −4,500 V (negative). The gas source I (GS I) was maintained at 55 psi, gas source II (GS II) at 60 psi, and curtain gas (CUR) at 25 psi. Collision-activated dissociation (CAD) parameters were set to high. In the triple quadrupole (QTRAP), each ion pair was scanned and detected based on optimized declustering potential (DP) and collision energy (CE).

### 2.8 Data analysis

All samples were subjected to three biological replicates, and the data are presented as mean ± standard deviation.

**Figure 1:**
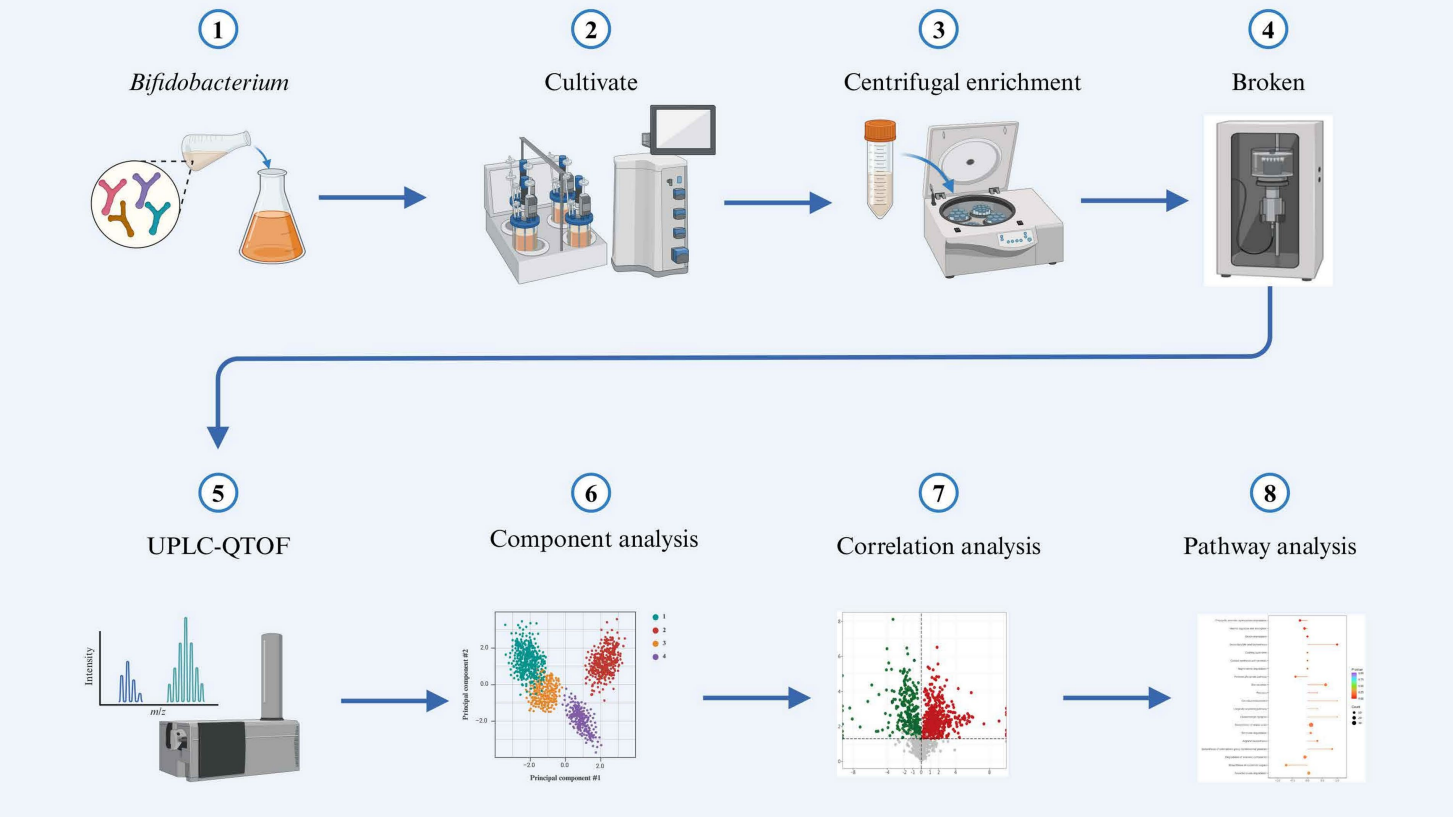
Schematic diagram illustrating the experimental workflow.

The mass spectra data underwent peak extraction, noise reduction, deconvolution, and peak alignment. Comprehensive library searching and identification were performed using the MassBank,^2^ ReSpect,^3^ and GNPS^4^ databases. Utilizing principles of chemometrics and multivariate statistics, the multidimensional data were subjected to dimensionality reduction and classification analysis. Principal component analysis (PCA) and orthogonal partial least squares discriminant analysis (OPLS-DA) of the three-dimensional data matrix were conducted using R software to identify differential metabolites. Hierarchical cluster analysis was also performed with R software, generating heatmaps (using the pheatmap package) to characterize the accumulation patterns of metabolites among samples (Chong and Xia, 2018). The identified compounds were further analyzed for metabolic pathways using the Kyoto Encyclopedia of Genes and Genomes (KEGG) database (Ogata et al., 1999).^5^ The sample preparation and analysis workflow are illustrated in Figure 1.

## 3 Results and discussion

### 3.1 Growth curves

**Figure 2:**
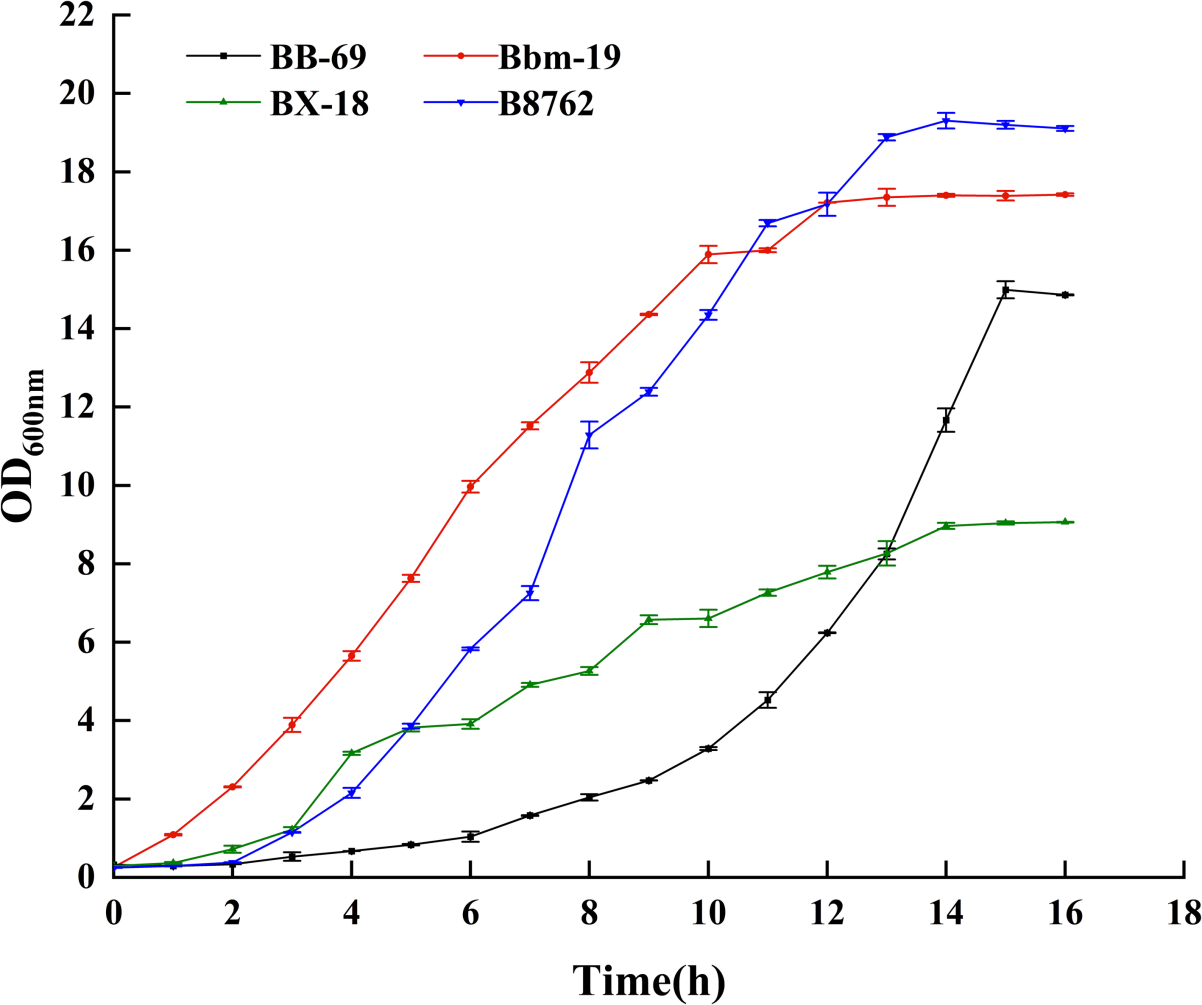
Growth curves of four strains of *Bifidobacteria*.

In this study, the absorbance value (OD_600nm_) was used to construct the growth curves of four strains of *Bifidobacterium*, we strictly controlled the sampling points to ensure the reliability and reproducibility of the data, and we performed three biological replicates at each time point, and the results were plotted as mean ± standard deviations, as can be seen from Figure 2 (Supplementary Table S3), BB-69 entered the stable phase at 15 h, Bbm-19 entered the stable phase at 12 h, BX-18 and B8762 entered the stable phase at 14 h, and the nutrients in the culture medium were gradually depleted, and the metabolites began to accumulate in large quantities. Therefore, the stable period was selected to harvest the bacteria for follow-up research.

### 3.2 Prebiotic properties of four strains of *Bifidobacteria*

We performed gastrointestinal fluid tolerance tests on Bbm-19 and BX-18. *Bifidobacteria* were inoculated in simulated gastrointestinal fluids, and their survival rate was monitored to assess their tolerance in the environment of gastric acid and bile salts. The specific results are as follows:

Bbm-19 also demonstrates good acid and bile salt tolerance, with a survival rate of 61.16% after 3 h in gastric juice at pH 2.5 and a survival rate of 95.09% after 8 h in intestinal fluid at pH 8.0.

BX-18 exhibits good acid and bile salt tolerance, with a survival rate of 42.02% after 3 h in gastric juice at pH 2.5 and a survival rate of 37.50% after 8 h in intestinal fluid at pH 8.0.

Previous research by the laboratory team has shown that: BB-69 exhibits good acid and bile salt tolerance, with a survival rate of 73.54% after 3 h in gastric juice at pH 2.5 and a survival rate of 91.29% after 8 h in intestinal fluid at pH 8.0. These physiological characteristics indicate that this strain possesses essential probiotic properties. Additionally, results from an animal study suggest that it alleviates symptoms of experimental colitis in mice through mechanisms such as enhancing the structure and function of the intestinal mucosal barrier, reducing oxidative stress damage in the intestine, inhibiting excessive immune activation, and lowering inflammation levels (Han et al., 2024).

B8762 also shows good acid and bile salt tolerance, with a survival rate of 61.26% after 3 h in gastric juice at pH 2.5 and a survival rate of 87.70% after 8 h in intestinal fluid at pH 8.0. Furthermore, a study indicates that both live *Bifidobacterium longum* subsp. infantis B8762 and heat-killed cells can alleviate DSS-induced colitis in rats (Li et al., 2024).

In summary, the four strains of *Bifidobacterium* have potential probiotic properties and growth characteristics, which provide basic research for the study of their physiological and metabolic properties.

### 3.3 Principal component analysis and orthogonal partial least squares discriminant analysis

A total of 12 samples from four bifidobacterial strains in triplicate were investigated using untargeted metabolomics analysis. To assess the intra- and intergroup statistical differences in total metabolites, the metabolomics data were first normalized, followed by multivariate statistical analysis, including PCA and OPLS-DA.

**Figure 3:**
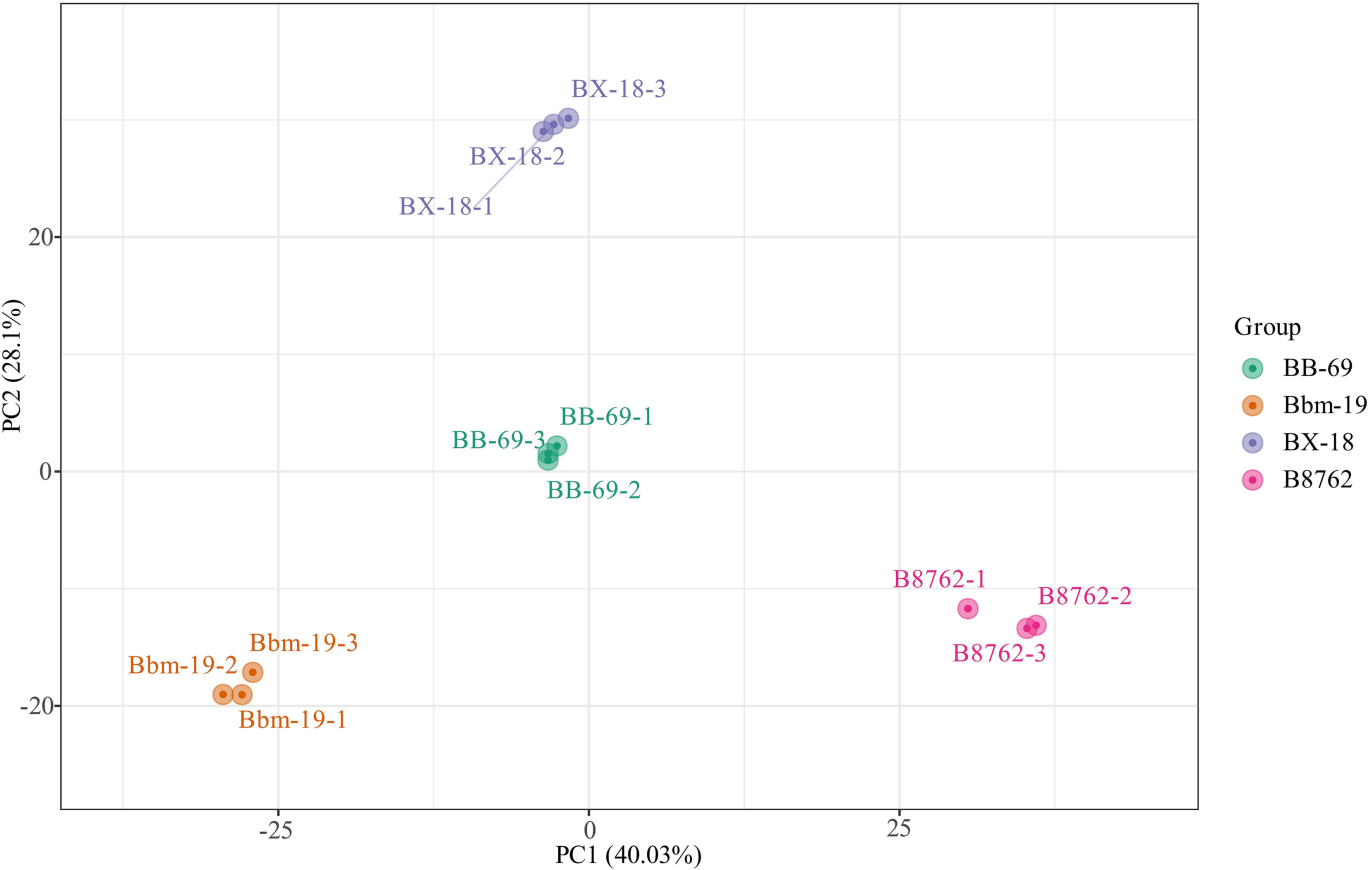
Principal component analysis score plot for the cell metabolomes of four bifidobacterial strains: *Bifidobacterium animalis* subsp. *lactis* BB-69, Bbm-19, *Bifidobacterium brevis* BX-18, and *Bifidobacterium longum* subsp. *infantis* B8762.

In the PCA, the symbols representing different samples showed distinct strain-based clustering patterns on the PCA score plot (Figure 3), with principal components PC1 and PC2 accounting for 40.03 and 28.10% of the total variance, respectively. Afterwards, OPLS-DA was employed to identify differential metabolites between bifidobacterial strains. The predictive parameters of the OPLS-DA model include *R*^2^*X*, *R*^2^*Y*, and *Q*^2^, where *R*^2^*X* and *R*^2^*Y* represent the explanatory rates of the model, respectively, and *Q*^2^ indicates the predictive ability of the model. Values closer to 1 for these indicators reflect a more stable and reliable model. A *Q*^2^ value greater than 0.5 is considered effective, while a value exceeding 0.9 is considered excellent (Zhang et al., 2015). In the OPLS-DA model, the pairwise comparisons between samples of BB-69, B8762, BX-18, and Bbm-19 were yielded high *Q*^2^ (0.984–0.994), *R*^2^*X* (0.809–0.852), and *R*^2^*Y* (range = 1; Supplementary Table S1), demonstrating high predictability and strong fitness of the models.

The S-plots of the pairwise comparisons by OPLS-DA are shown in Figure 3. A total of 1,340 metabolites (B8762 vs. BX-18), 1,340 metabolites (B8762 vs. Bbm-19), 1,340 metabolites (BX-18 vs. BB-69), 1,338 metabolites (B8762 vs. Bbm-19), 1,339 metabolites (BX-18 vs. Bbm-19), and 1,338 metabolites (BB-69 vs. Bbm-19) displayed VIP values greater than 1, and were positioned furthest from the origin. Therefore, these metabolites can serve as biomarkers for distinguishing different *Bifidobacterium* strains.

**Figure 4:**
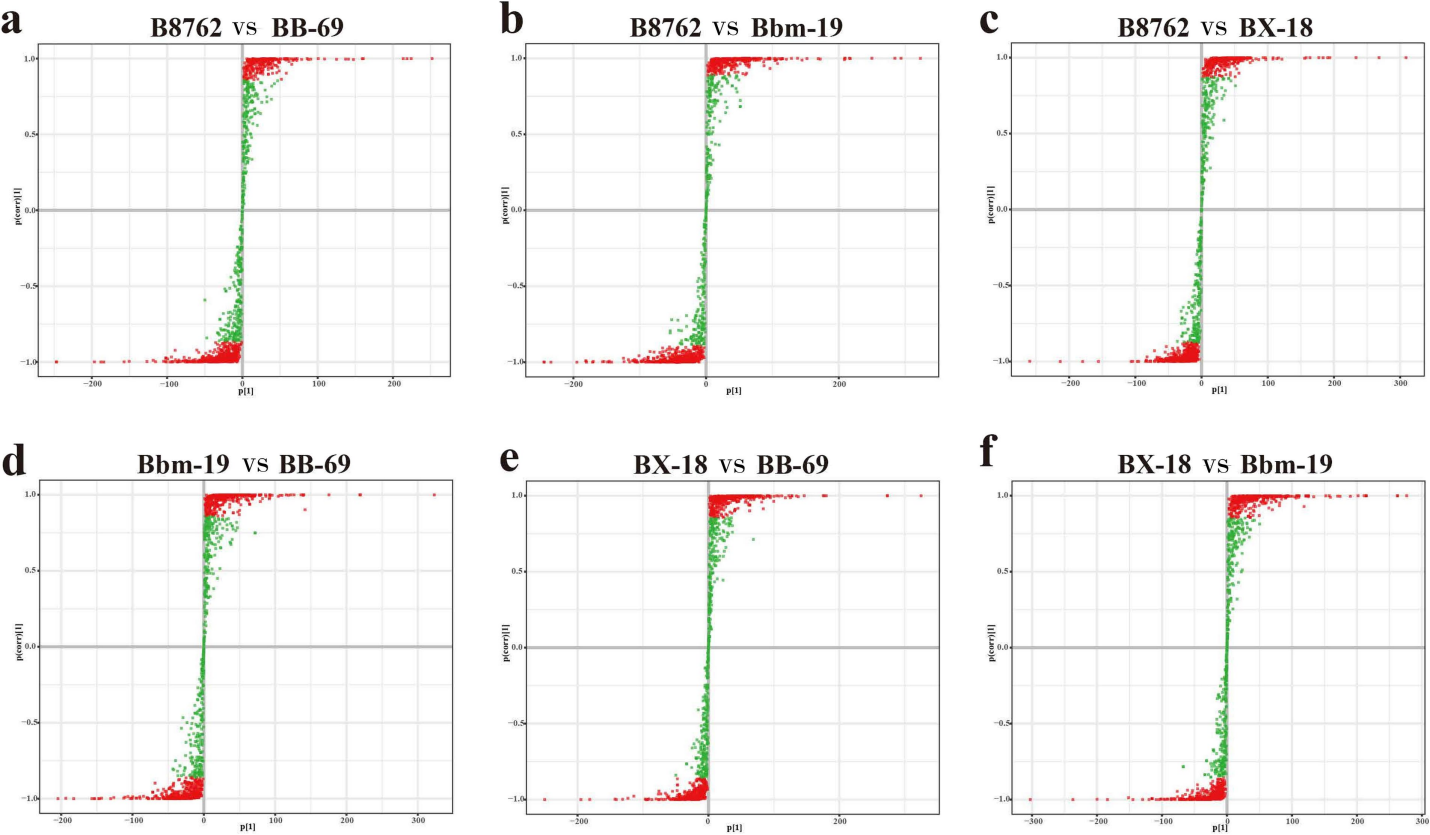
S-plot of pairwise comparisons among bifidobacterial cell metabolomes using orthogonal partial least squares discriminant analysis. The comparisons include: **(A)** B8762 vs. BB-69, **(B)** B8762 vs. Bbm-19, **(C)** B8762 vs. BX-18, **(D)** Bbm-19 vs. BB-69, **(E)** BX-18 vs. BB-69, and **(F)** BX-18 vs. Bbm-19. Each S-plot illustrates the relationship between metabolites and principal components, with covariance plotted on the *x*-axis and correlation coefficients on the *y*-axis, providing a visual representation of specific metabolites. Metabolites located in the upper right and lower left corners indicate greater differences between strains. Metabolites with a variable importance in projection (VIP) score greater than 1 are represented by red dots, while those with a VIP score of 1 or less are shown as green dots.

**Figure 5:**
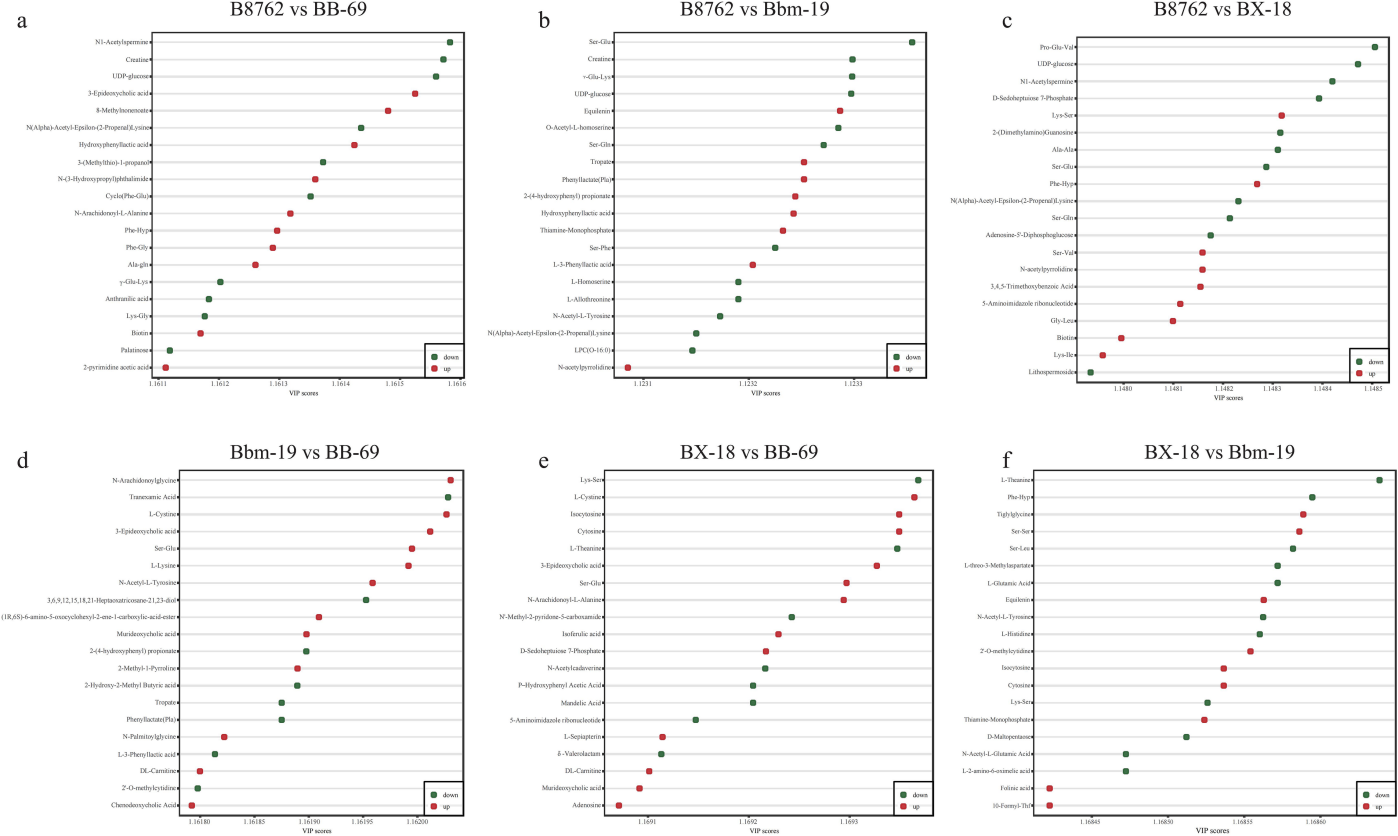
Top 20 metabolites with the highest variable importance in projection (VIP) scores for each pairwise comparison of bifidobacterial strains. The comparisons include: **(A)** B8762 vs. BB-69, **(B)** B8762 vs. Bbm-19, **(C)** B8762 vs. BX-18, **(D)** Bbm-19 vs. BB-69, **(E)** BX-18 vs. BB-69, and **(F)** BX-18 vs. Bbm-19. Red dots indicate metabolites with increased levels, while green dots represent those with decreased levels.

The top 20 differential metabolites with the highest VIP values for each strain are presented in Figure 4 (Croux et al., 2000). In the comparison between B8762 and BB-69, notable metabolites included 3-epideoxycholic acid, 8-methylnonenoate, and hydroxyphenyllactic acid (Figure 5A). For the B8762 and Bbm-19 comparison, key metabolites were equilenin, tropate, and phenyllactate (Figure 5B). In the B8762 and BX-18 comparison, significant metabolites included Lys-Ser, Phe-Hyp, and Ser-Val (Figure 5C). The comparison between Bbm-19 and BB-69 highlighted N-arachidonoylglycine, L-cystine, and 3-epideoxycholic acid (Figure 5D). In the BX-18 and BB-69 comparison, the main metabolites were L-cystine, isocytosine, and cytosine (Figure 5E), while the BX-18 and Bbm-19 comparison revealed tiglylglycine, equilenin, and 2-O-methylcytidine (Figure 5F).

**Figure 6:**
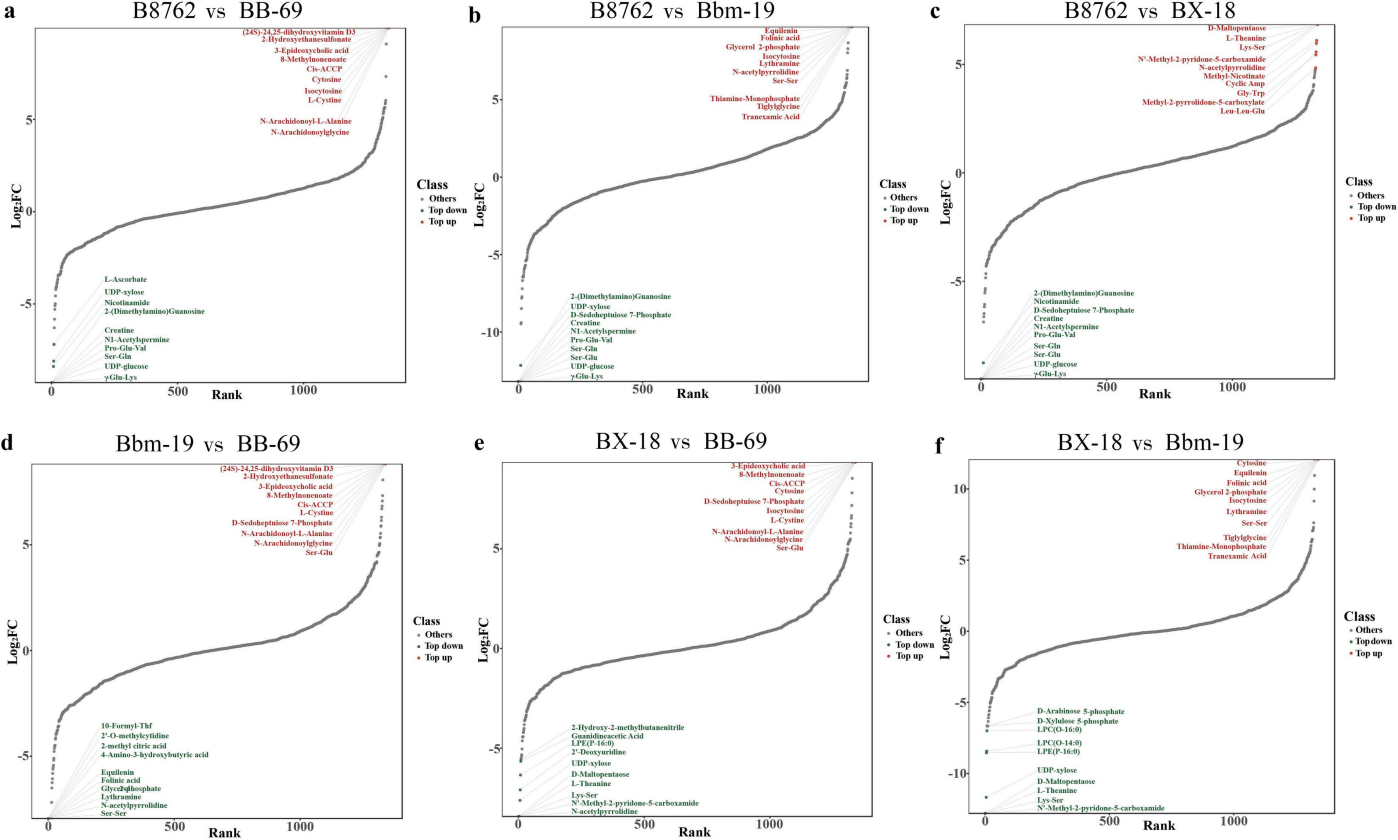
Dynamic distribution plots generated by pairwise comparisons of bifidobacterial cell metabolomes. The comparisons include: **(A)** B8762 vs. BB-69, **(B)** B8762 vs. Bbm-19, **(C)** B8762 vs. BX-18, **(D)** Bbm-19 vs. BB-69, **(E)** BX-18 vs. BB-69, and **(F)** BX-18 vs. Bbm-19. The *x*-axis displays the cumulative count of metabolites, sorted from the smallest to the largest fold change (FC), while the *y*-axis represents the log_2_(FC). Each dot corresponds to a metabolite, with green dots indicating the top 10 decreased metabolites and red dots representing the top 10 increased metabolites. These plots effectively highlight the most significantly altered metabolites.

We then created dynamic plots of differential metabolite distributions to illustrate metabolites of greatest differences between compared samples (Figure 6). In the comparison between B8762 and BB-69, the metabolite with the highest fold change (FC) was (24S)-24,25-dihydroxyvitamin D3, while the one with the lowest FC was Glu-Val. For B8762 versus BX-18, the highest FC was observed in D-maltopentaose, and the lowest in Ser-Gln. In the B8762 and Bbm-19 comparison, equilenin exhibited the highest FC, with Ser-Gln having the lowest. In the BX-18 versus BB-69 comparison, 3-epideoxycholic acid showed the highest FC, while L-theanine had the lowest. For BX-18 compared to Bbm-19, For BX-18 compared to Bbm-19, cytosine had the highest FC, and D-maltopentaose had the lowest. Finally, in the Bbm-19 versus BB-69 comparison, (24S)-24,25-Dihydroxyvitamin D3 again showed the highest FC, with glycerol 2-phosphate having the lowest.

### 3.4 Differential metabolite screening

**Figure 7:**
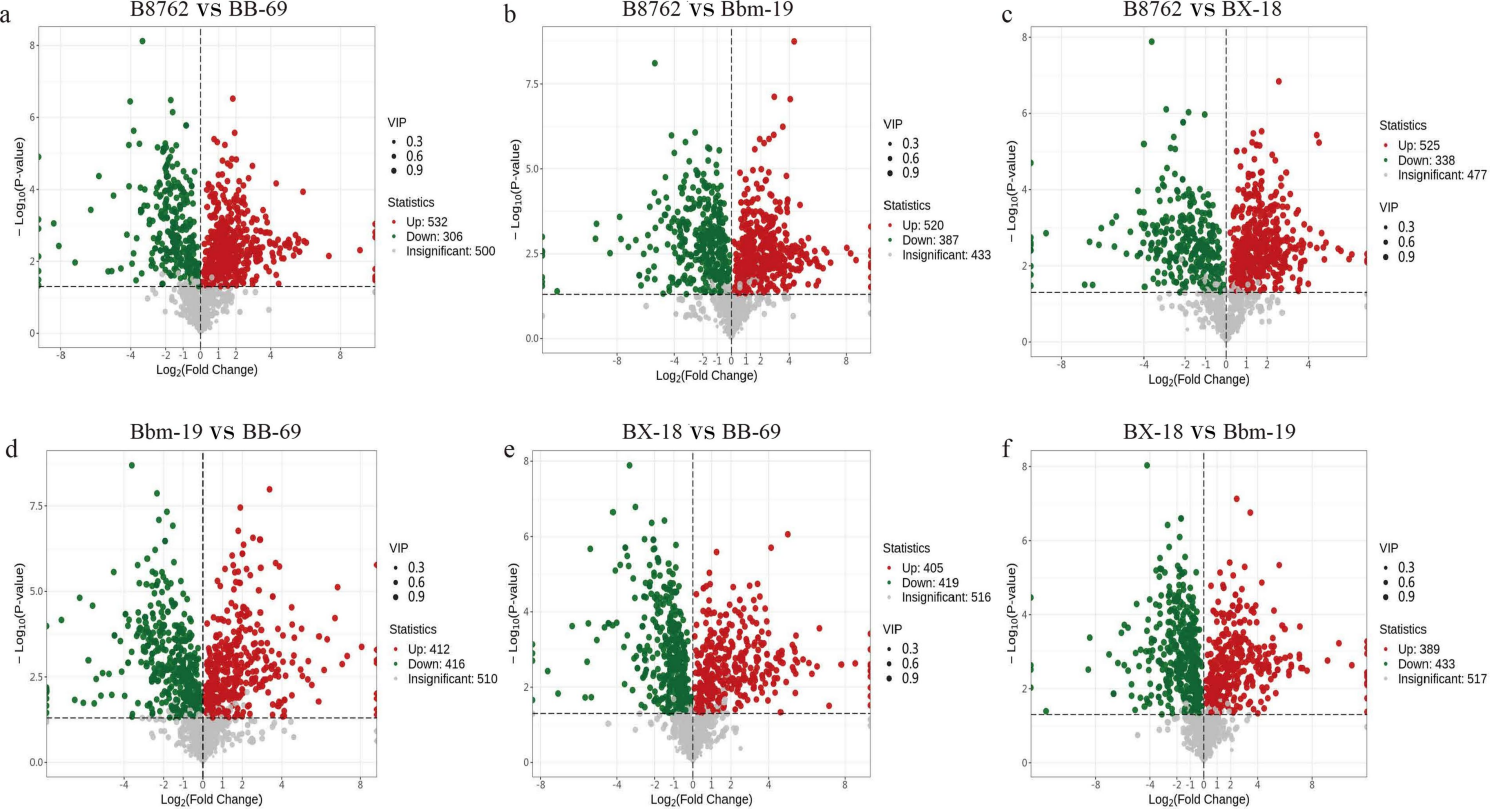
Volcano plots showing significant differential metabolites from pairwise comparisons of bifidobacterial cell metabolomes. The comparisons include: **(A)** B8762 vs. BB-69, **(B)** B8762 vs. Bbm-19, **(C)** B8762 vs. BX-18, **(D)** Bbm-19 vs. BB-69, **(E)** BX-18 vs. BB-69, and **(F)** BX-18 vs. Bbm-19. In these plots, red dots indicate significantly increased metabolites, green dots represent significantly decreased metabolites, and gray dots denote metabolites with no significant change.

To gain deeper insights into the metabolic differences among the four bifidobacterial strains, we screened differential metabolites from a total of 1,340 metabolites (VIP value >1) based on the threshold of FC ≥2 or ≤0.5 and a *p*-value <0.05 from the t-test. The results are displayed in volcano plots (Figure 7). The number of significantly different metabolites identified between pairwise comparisons: in the comparison between B8762 and BB-69, 838 significantly different metabolites were detected, with 532 upregulated and 306 downregulated; in the B8762 and Bbm-19 comparison, 907 significantly different metabolites were identified, including 520 upregulated and 387 downregulated; in the B8762 versus BX-18 comparison, 863 significantly different metabolites were found, with 525 upregulated and 338 downregulated; in the Bbm-19 versus BB-69 comparison, 828 significantly different metabolites were identified, with 412 upregulated and 416 downregulated; for the BX-18 versus BB-69 comparison, 824 significantly different metabolites were noted, with 405 upregulated and 419 downregulated; and in the BX-18 versus Bbm-19 comparison, 822 significantly different metabolites were found, consisting of 389 upregulated and 433 downregulated. These findings provide important information for further functional analysis and exploration of metabolic pathways.

### 3.5 Metabolite hierarchical clustering analysis

**Figure 8:**
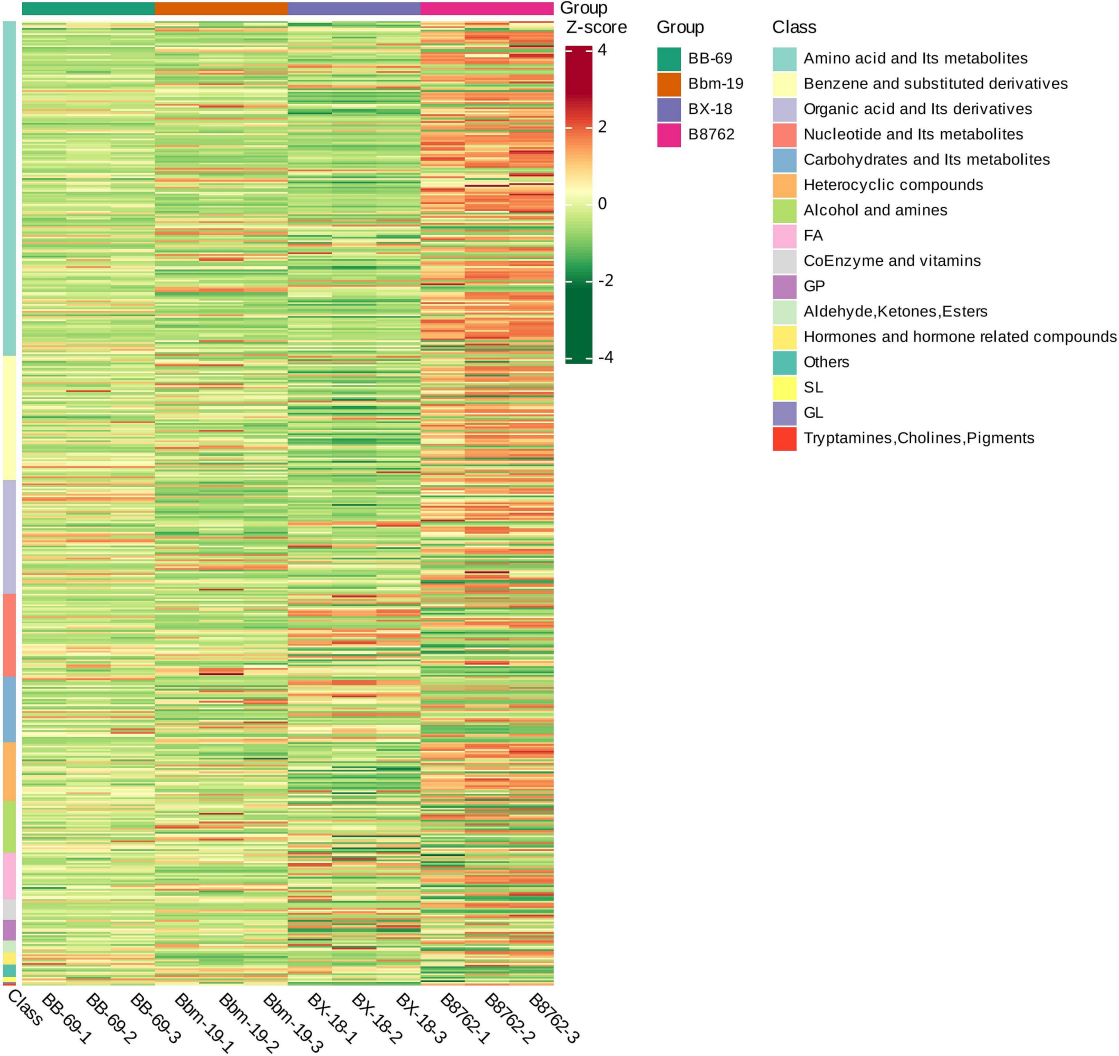
Heatmap of differential metabolite clustering. This heatmap visually illustrates the variations in differential metabolite profiles, derived from the original metabolomics data processed using the unit variance scaling method. The horizontal axis represents the different strains: *Bifidobacterium animalis* subsp. *lactis* BB-69, Bbm-19, *Bifidobacterium brevis* BX-18, and *Bifidobacterium longum* subsp. *infantis* B8762 (each represented in triplicate, with suffix numbers indicating sample replicates). The vertical axis displays the biochemical classes of the differential metabolites. Color coding within the heatmap indicates relative abundance levels after standardization, with red representing high abundance (high *Z*-score) and green indicating low abundance (low *Z*-score).

To visually represent the variations in differential metabolite profiles by biochemical classes, the original data for the selected metabolites were processed using the unit variance scaling method and were subjected to hierarchical clustering analysis, with results displayed in a heatmap (Figure 8). The level in the figure indicates the name of the sample, the longitudinal information is the differential metabolite, the red represents the concentration of the high-content substance, and the green represents the concentration of the low-content substance.

The differential metabolites among the four bifidobacterial strains primarily belonged to the biochemical classes of amino acids and their metabolites, benzene and substituted derivatives, organic acids and their derivatives, nucleotides and their metabolites, carbohydrates and their metabolites, heterocyclic compounds, alcohols and amines, fatty acids, coenzymes and vitamins, glycerophospholipids, aldehydes, ketones, and esters, and hormones and hormone-related compounds. Notably, the distribution of biochemical class of differential metabolites exhibited substantial variations between strains.

### 3.6 Differential metabolite K-means analysis

**Figure 9:**
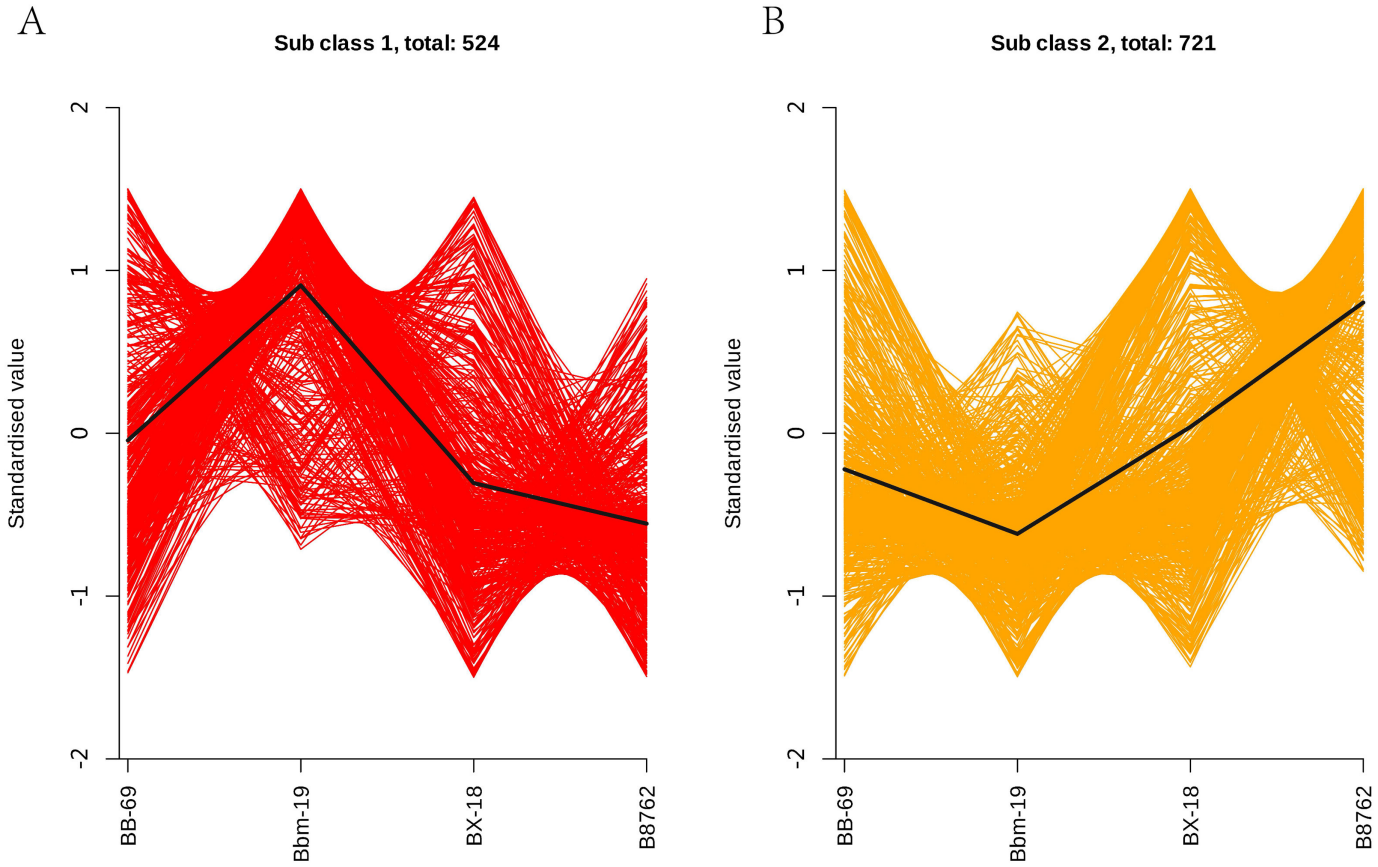
K-means clustering analysis of bifidobacterial cell metabolomes. Four strains were analyzed: *Bifidobacterium animalis* subsp. *lactis* BB-69, Bbm-19, *Bifidobacterium brevis* BX-18, and *Bifidobacterium longum* subsp. *infantis* B8762. This analysis identified two distinct clusters, with the standardized scores for clusters 1 and 2 presented in panels **(A,B)**, respectively.

In order to study the trend of the relative content of metabolites in different groups, the relative contents of all differential metabolites identified according to the screening criteria in all comparison groups were treated with UV (unit variance scaling) (the mean of the original data minus the mean of the variable and divided by the standard deviation of the variable), and then the K-means cluster analysis was performed. The abscissa represents the sample grouping, the ordinate represents the normalized relative amount of metabolites, the subclass represents the metabolite class number of the same trend, and total: represents the number of metabolites in the class. The results indicated that the trends in these metabolite changes could be categorized into two distinct clusters. In cluster 1, a total of 524 metabolites were ranked in the following order of abundance: Bbm-19 > BB-69 > BX-18 > B8762 (Figure 9A). Conversely, in cluster 2, the order for the remaining 721 metabolites was: B8762 > BX-18 > BB-69 > Bbm-19 (Figure 9B).

### 3.7 Differential metabolomics profiles among four bifidobacterial strains

#### 3.7.1 Differential active metabolites in four *Bifidobacterium* strains

The analysis identified a total of 1,245 differential metabolites, categorized into various biochemical classes: include 68 types of alcohols and amines, 23 types of aldehydes, ketones, and esters, 429 types of amino acids and their metabolites, 141 types of benzene and substituted derivatives, 15 types of bile acids, 76 types of carbohydrates and their metabolites, 25 types of coenzymes and vitamins, 88 types of fatty acids, 3 types of glycolipids, 48 types of glycerophospholipids, 24 types of hormones and hormone-related compounds, 80 types of heterocyclic compounds, 186 types of organic acids and their derivatives, 5 types of sphingolipids, and 6 types of tryptamines, cholines, and pigments. Major results are summarized in Supplementary Table S2.

##### 3.7.1.1 Alcohols and amines

Among the four strains, Bbm-19 showed the highest diversity in alcohols and amines, with 31 distinct types, while B8762 had the fewest. Notably, phenethylamine, a biogenic amine and monoamine neurotransmitter, was particularly abundant among the 31 metabolites. This metabolite has been reported to increase dopamine levels in the extracellular fluid while inhibiting dopamine neural activation, making it a potential treatment for depression (Xu et al., 1992).

##### 3.7.1.2 Amino acids and their metabolites

In Bbm-19, the most abundant category was amino acids and their metabolites. One significant metabolite was L-citrulline, recognized for its ability to enhance vasodilation and lower blood pressure (Ochiai et al., 2012). Another significant metabolite was L-arginine, a critical amino acid playing an essential role in infant growth and development and is vital for the urea cycle (Creager et al., 1992). It converts ammonia to urea and thereby reducing blood ammonia levels (Nan et al., 2006). In BX-18, a prominent differential abundant metabolite was L-cystathionine, is noteworthy for its cardiovascular protective effects (Nan et al., 2006). In B8762, a noteworthy differential abundant metabolite was L-carnosine, which exhibits strong antioxidant abilities, effectively scavenging reactive oxygen species generated during oxidative stress (Kim et al., 2011), a function not observed in the other strains. Another differential abundant metabolite was L-glutamine, a non-essential amino acid that can be synthesized from glucose, is important for protein synthesis and has therapeutic applications in treating gastric and duodenal ulcers, gastritis, and hyperacidity, as well as enhancing brain function (Li et al., 1998). Moreover, differentially abundant N-acetylcysteine was observed, which is a scavenger of reactive oxygen species and an antiviral metabolite against influenza viruses (Behr, 2012). Conversely, BB-69 displays a lower diversity of bioactive metabolites within the amino acids and their metabolites category.

##### 3.7.1.3 Bile acids

Among the four strains, B8762 exhibits a greater variety of bile acids, such as ketolithocholic acid, which is known to be absorbed by the body and can suppress endogenous bile acid production as well as biliary cholesterol secretion (Choucair et al., 2020). Bbm-19 showed significantly higher levels of deoxycholic acid compared to the other three groups. Deoxycholic acid, a by-product of intestinal metabolism, is recognized for its ability to activate the G protein-coupled bile acid receptor TGR5, which plays a crucial role in metabolic regulation (Bala et al., 2014).

##### 3.7.1.4 Coenzymes and vitamins

Coenzymes and vitamins were most abundant in BX-18, followed by BB-69. Notably, 4-hydroxyretinoic acid was only present in BX-18; this naturally occurring derivative of retinoic acid has been shown to participate in various physiological processes, including immune regulation, neuroprotection, and antioxidant effects (Zhou et al., 2023). In BB-69, L-ascorbate and pantothenate were exclusively present. L-ascorbate is an endogenous antioxidant and exhibits anticancer properties by generating reactive oxygen species that selectively target and damage cancer cells (Huang et al., 2012). Pantothenate, an essential micronutrient, serves as a precursor to coenzyme A, playing a crucial role in regulating carbohydrate, lipid, protein, and nucleic acid metabolism (Cronan, 2023). Nicotinamide was exclusively present in Bbm-19, where it has demonstrated beneficial effects in treating rough skin and oral inflammation, delaying skin aging, inhibiting melanin deposition, and promoting skin whitening (Bitterman et al., 2002). Pyridoxine was unique to B8762, with its deficiency linked to cardiovascular and neurological problems (Andac-Ozturk et al., 2023).

##### 3.7.1.5 Organic acids and their derivatives

The category of organic acids and their derivatives was most abundant in BB-69, followed by B8762 and Bbm-19, while BX-18 contained the fewest types. Cinnamic acid was present in both B8762 and BB-69, which shows potential for cancer intervention (Liu et al., 1995). Traumatin, co-present in BB-69 and Bbm-19, is recognized for its wound healing properties, enhancing collagen biosynthesis in cultured human skin fibroblasts. Additionally, it can inhibit the survival of MCF-7 breast cancer cells, enhancing apoptosis and oxidative stress, which positions it as a valuable compound for research into cancer, hematological disorders (including arterial hypertension), and skin diseases related to oxidative stress and collagen biosynthesis disorders (Güneş et al., 2015).

The four strains of bifidobacterial strains contain varying amounts and types of active metabolites. BB-69 has a greater variety of organic acids and derivatives, while Bbm-19 has a higher quantity of amino acids and metabolites. BX-18 has a greater variety of coenzymes and vitamins, and B8762 has a larger variety of bile acids. This diversity in active metabolites reflects their physiological variations, which allows for more choices for in industrial production of probiotics or functional products derived from single strains or combinations of multiple strains.

#### 3.7.2 Differential metabolite KEGG enrichment analysis

Many bifidobacterial strains are considered probiotics and are often cultivated using high-density cultivation techniques. Understanding their metabolic profile is essential for optimizing their laboratory-based and industrial cultivation. To explore the metabolic pathways associated with the differential metabolites, we annotated them utilizing the KEGG database, which further enables us to conduct KEGG pathway enrichment analysis.

**Figure 10:**
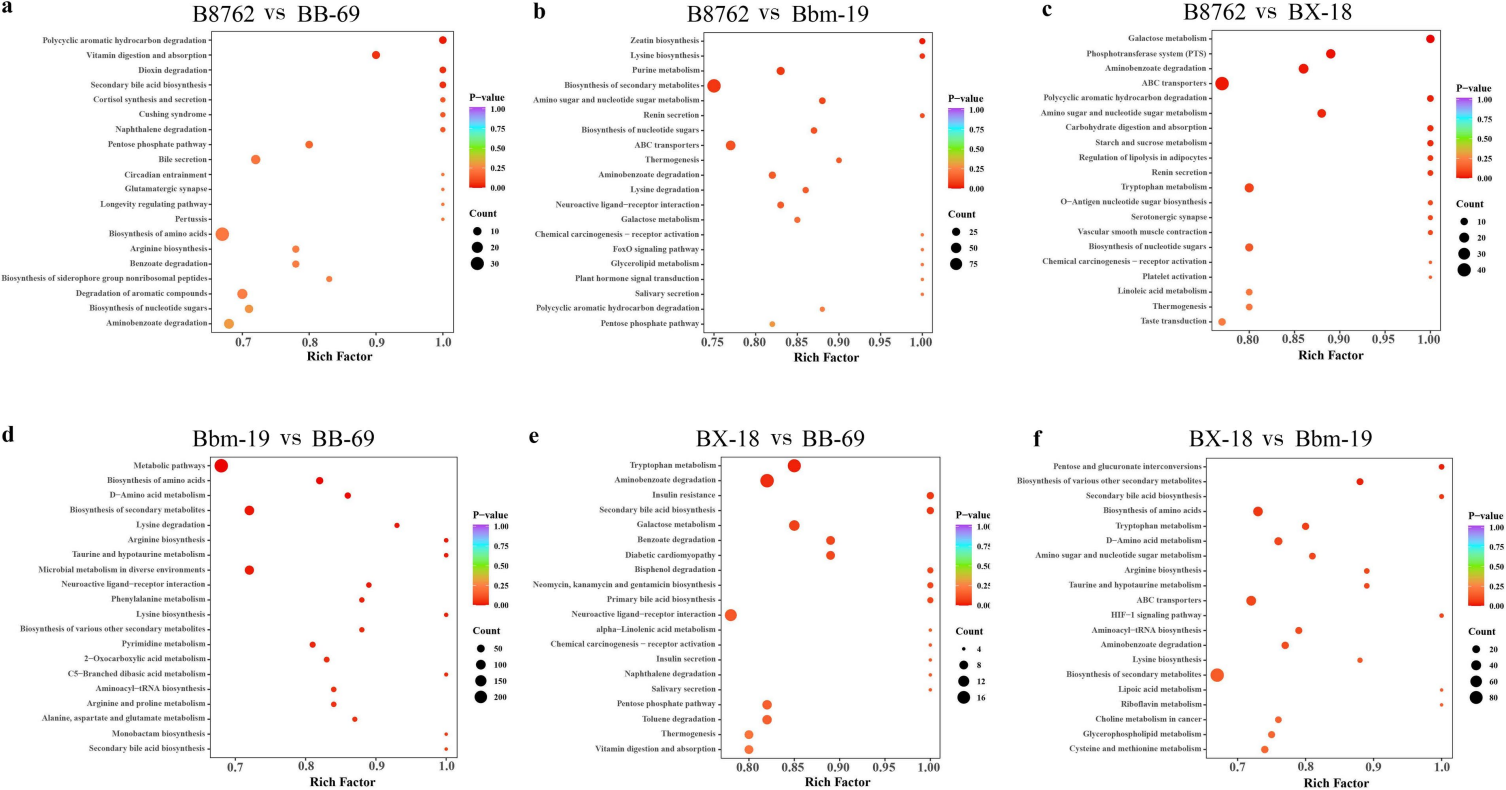
Kyoto Encyclopedia of Genes and Genomes (KEGG) annotation and pathway enrichment analysis based on differentially abundant metabolites. The comparisons include: **(A)** B8762 vs. BB-69, **(B)** B8762 vs. Bbm-19, **(C)** B8762 vs. BX-18, **(D)** Bbm-19 vs. BB-69, **(E)** BX-18 vs. BB-69, and **(F)** BX-18 vs. Bbm-19. The *x*-axis displays the rich factor for each pathway, while the *y*-axis lists the pathway names sorted by *p*-value. The color of the dots indicates the magnitude of the *p*-value, with more intense colors reflecting greater enrichment. Additionally, the size of the dots corresponds to the number of differential metabolites enriched within that pathway.

The top 20 metabolic pathways with the smallest *p*-values identified through KEGG analysis are presented (Figure 10). In the comparison between B8762 and BB-69, polycyclic aromatic hydrocarbons were found to be significantly enriched (*p* = 0.017). Other significantly enriched pathways included vitamin digestion and absorption (*p* = 0.048), secondary bile acid biosynthesis (*p* = 0.048), and dioxin degradation (*p* = 0.048). When comparing B8762 and Bbm-19 groups, the pathway of zeatin biosynthesis was significantly enriched (*p* = 0.017). In the analysis between B8762 and BX-18, galactose metabolism was significantly enriched (*p* = 0.002). Other significantly enriched pathways included phosphotransferase system (*p* = 0.0108), aminobenzoate degradation (*p*-value = 0.014), and ATP-binding cassette transporters (*p* = 0.014). In the comparison between Bbm-19 and BB-69, several metabolic pathways were significantly enriched, including metabolic pathways (*p* = 0.002), biosynthesis of amino acids (*p* = 0.004), and D-amino acid metabolism (*p* = 0.006). Other significantly enriched pathways in this comparison include biosynthesis of secondary metabolites (*p* = 0.014), lysine degradation (*p* = 0.016), and arginine biosynthesis (*p* = 0.017). In BX-18 and BB-69 comparison, tryptophan metabolism (*p* = 0.024) and aminobenzoate degradation (*p* = 0.040) were significantly enriched. Finally, in the comparison between BX-18 and Bnm-19, significant enrichments were observed in pentose and glucuronate interconversions (*p* = 0.022) and biosynthesis of various other secondary metabolites (*p* = 0.027).

#### 3.7.3 Differences in KEGG metabolic pathways

**Figure 11:**
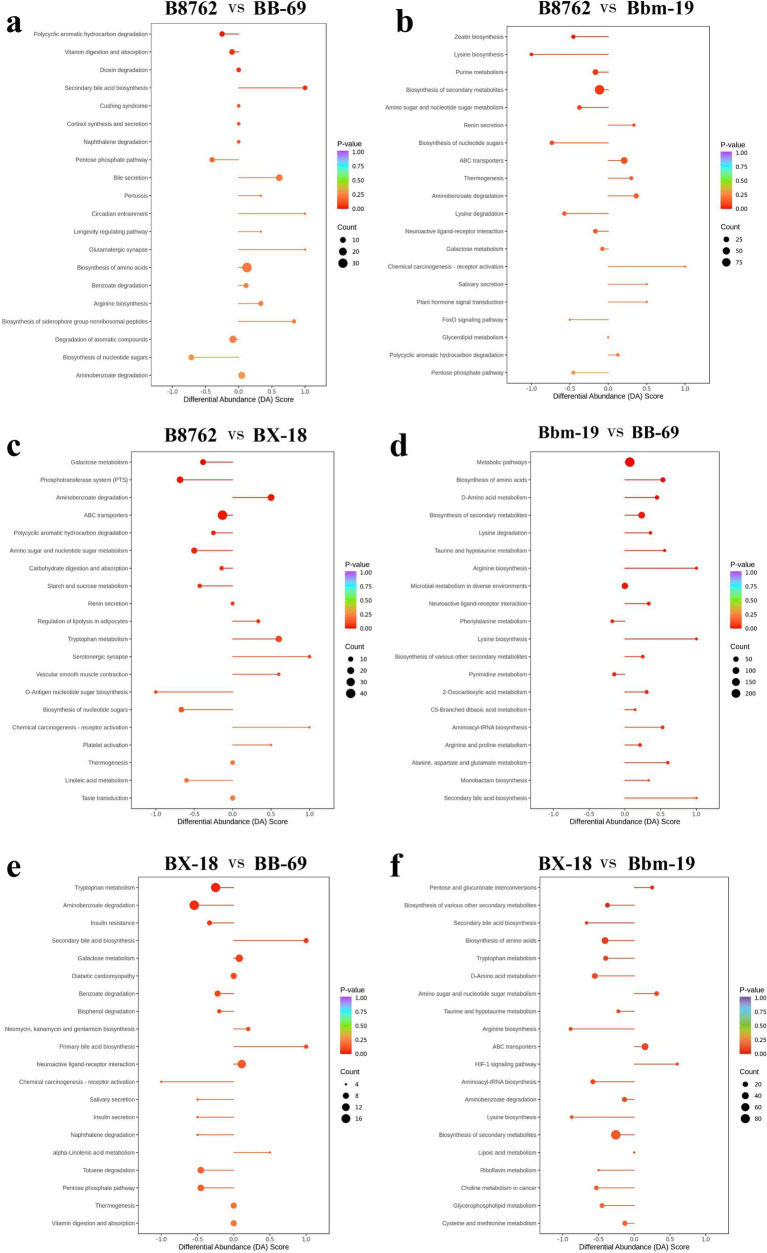
Differential abundance (DA) score plots of top differentially abundant metabolites from pairwise comparisons of bifidobacterial cell metabolomes. The comparisons include: **(A)** B8762 vs. BB-69, **(B)** B8762 vs. Bbm-19, **(C)** B8762 vs. BX-18, **(D)** Bbm-19 vs. BB-69, **(E)** BX-18 vs. BB-69, and **(F)** BX-18 vs. Bbm-19. The DA score quantifies overall changes in all metabolites within the metabolic pathways. A DA score of 1 indicates that all metabolites in the pathway are increased, while a score of −1 signifies that all metabolites are decreased. The length of the line segment represents the absolute value of the DA score, and the size of the circular endpoint indicates the number of differential metabolites within that pathway. The color of the line segments and circular points represents the magnitude of the *p*-value, with red indicating values closer to 0 and purple representing larger values, thereby highlighting differences in statistical significance.

Subsequently, we assessed the variation between KEGG metabolic pathways based on calculating the differential abundance (DA) score, which measures the overall changes in all metabolites within metabolic pathways (Figure 11).

In the comparison between B8762 and BB-69, pathways such as secondary bile acid biosynthesis, circadian entrainment, glutamatergic synapse, bile secretion, and biosynthesis of siderophore group nonribosomal peptides exhibited higher DA scores, indicating enhanced expression in these pathways in BB-69. Conversely, pathways including biosynthesis of nucleotide sugars, pentose phosphate pathway, and polycyclic aromatic hydrocarbon degradation showed lower DA scores, suggesting greater expression in B8762.

When comparing B8762 and Bbm-19, pathways including chemical carcinogenesis-receptor activation, renin secretion, salivary secretion, and plant hormone signal transduction displayed higher DA scores, indicating elevated expression in Bbm-19. In contrast, lysine biosynthesis, biosynthesis of nucleotide sugars, FoxO signaling pathway, and pentose phosphate pathway had lower DA scores, reflecting their higher expression in B8762.

In the analysis between B8762 and BX-18, pathways such as aminobenzoate degradation, tryptophan metabolism, serotonergic synapse, vascular smooth muscle contraction, and chemical carcinogenesis-receptor activation had higher DA scores, indicating increased expression in BX-18. On the other hand, pathways like galactose metabolism, phosphotransferase system, O-antigen nucleotide sugar biosynthesis, biosynthesis of nucleotide sugars, and linoleic acid metabolism had lower DA scores, suggesting higher expression in B8762.

In the comparison between Bbm-19 and BB-69, pathways such as phenylalanine metabolism and pyrimidine metabolism exhibited lower DA scores, indicating higher expression in BB-69. Other pathways, including bisosynthesis of amino acids, D-amino acid metabolism, lysine degradation and so on showed higher DA scores, indicating higher expression in Bbm-19.

For the comparison between BX-18 and BB-69, pathways such as secondary bile acid biosynthesis, primary bile acid biosynthesis, and alpha-linolenic acid metabolism displayed higher DA scores, suggesting increased expression in BB-69. In contrast, other pathways, including trypophan metabolism, aminobenzoate degradation, insulin resistance and so on, had lower DA scores, indicating higher expression in BX-18.

Lastly, in the comparison between BX-18 and Bbm-19, pathways including pentose and glucuronate interconversions, amino sugar and nucleotide sugar metabolism, ABC transporters, and HIF-1 signaling pathway showed higher DA scores, indicating elevated expression in Bbm-19. Conversely, other pathways, including secondary bile acid biosynthesis, bisosynthesis of amino acids, aryginine biosynthesis and so on had lower DA scores, suggesting higher expression in BX-18.

In summary, this study identified several differentially enriched pathways among the four bifidobacterial strains, offering valuable insights into their metabolic flexibility, functional diversity, and ecological roles. These findings highlight the varied contributions of bifidobacteria to health and disease, laying the groundwork for future research and the potential application of these microorganisms in developing functional health products.

## 4 Conclusion

The study analyzed the metabolic profiles of four bifidobacterial strains using a non-targeted metabolomics approach, identifying various metabolites, including alcohol, amines, aldehyde, ketones, esters, amino acids, benzene, bile acids, carbohydrates, coenzymes, vitamins, fatty acids, glycerophospholipids, hormones, heterocyclic compounds, organic acids, sphingolipids, tryptamines, cholines, and pigments. The studied strains exhibit distinct intracellular metabolic profiles and pathways, which may influence their physiology and host health benefits. BB-69 contains a greater variety of organic acids and derivatives, while Bbm-19 primarily features amino acids and metabolites. BX-18 has a greater variety of coenzymes and vitamins, while B8762 has a larger variety of bile acids. These metabolic differences provide a basis for developing functional foods, probiotic supplements, personalized nutrition, and precision medicine. Future research should explore how these metabolic differences affect host health and their potential applications in food science and medical health. The study enhances our understanding of the metabolic diversity of bifidobacteria, offering new strategies for improving human health.

## Data Availability

The raw data supporting the conclusions of this article will be made available by the authors, without undue reservation.

## Footnotes

^1^ https://www.imhpc.com/iLABdb

^2^ http://www.massbank.jp/

^3^ http://spectra.psc.riken.jp/

^4^ https://gnps.ucsd.edu/

^5^ https://www.genome.jp/kegg/pathway.html

## References

[ref17] Andac-Ozturk S. Garipoğlu G. Çatak J. (2023). Investigation of the vitamins B1, B2, and B6 vitamers bioaccessibilities of canned, dried legumes after in vitro gastrointestinal digestion system. Food Res. Int. 160. doi: 10.1016/j.foodres.2022.111671 36076445

[ref1] Bala V. Rajagopal S. Kumar D. P. Nalli A. D. Mahavadi S. Sanyal A. J.. (2014). Release of GLP-1 and PYY in response to the activation of G protein-coupled bile acid receptor TGR5 is mediated by Epac/PLC-ε pathway and modulated by endogenous H2S. Front. Physiol. 5:420. doi: 10.3389/fphys.2014.00420, PMID: 25404917 PMC4217307

[ref2] Behr J. (2012). Prednisone, azathioprine, and N-acetylcysteine for pulmonary fibrosis. N. Engl. J. Med. 367, 869–871. doi: 10.1056/NEJMc1207471 22931324

[ref3] Bezkorovainy A. (2020). Biochemistry and physiology of *Bifidobacterium*. Boca Raton, FL: CRC Press.

[ref4] Bitterman K. J. Anderson R. M. Cohen H. Y. Latorre-Esteves M. Sinclair D. A. (2002). Inhibition of silencing and accelerated aging by nicotinamide, a putative negative regulator of yeast sir2 and human SIRT1. J. Biol. Chem. 277, 45099–45107. doi: 10.1074/jbc.M205670200, PMID: 12297502

[ref5] Chen W. Gong L. Guo Z. Wang W. Zhang H. Liu X.. (2013). A novel integrated method for large-scale detection, identification, and quantification of widely targeted metabolites: application in the study of rice metabolomics. Mol. Plant 6, 1769–1780. doi: 10.1093/mp/sst080, PMID: 23702596

[ref6] Chen Q. W. Qiao J. Y. Cao M. W. Han Z. Y. Zeng X. Zhang X. Z. (2023). Spore germinator-loaded polysaccharide microspheres ameliorate colonic inflammation and tumorigenesis through remodeling gut microenvironment. Mater. Today 63, 32–49. doi: 10.1016/j.mattod.2023.02.002

[ref7] Chong J. Xia J. (2018). MetaboAnalyst R: an R package for flexible and reproducible analysis of metabolomics data. Bioinformatics 34, 4313–4314. doi: 10.1093/bioinformatics/bty528, PMID: 29955821 PMC6289126

[ref8] Choucair I. Nemet I. Li L. Cole M. A. Skye S. M. Kirsop J. D.. (2020). Quantification of bile acids: a mass spectrometry platform for studying gut microbe connection to metabolic diseases. J. Lipid Res. 61, 159–177. doi: 10.1194/jlr.RA119000311, PMID: 31818878 PMC6997600

[ref9] Creager M. A. Gallagher S. J. Girerd X. J. Coleman S. M. Dzau V. J. Cooke J. P. (1992). L-arginine improves endothelium-dependent vasodilation in hypercholesterolemic humans. J. Clin. Invest. 90, 1248–1253. doi: 10.1172/JCI115987, PMID: 1401062 PMC443166

[ref10] Creydt M. Hudzik D. Rurik M. Kohlbacher O. Fischer M. (2018). Food authentication: small-molecule profiling as a tool for the geographic discrimination of German white asparagus. J. Agric. Food Chem. 66, 13328–13339. doi: 10.1021/acs.jafc.8b05791, PMID: 30472843

[ref11] Creydt M. Wegner B. Gnauck A. Hörner R. Hummert C. Fischer M. (2022). Food authentication in the routine laboratory: determination of the geographical origin of white asparagus using a simple targeted LC-ESI-QqQ-MS/MS approach. Food Control 135:108690. doi: 10.1016/j.foodcont.2021.108690

[ref12] Cronan J. E. (2023). How an overlooked gene in coenzyme a synthesis solved an enzyme mechanism predicament. Mol. Microbiol. 119, 687–694. doi: 10.1111/mmi.15070, PMID: 37140060 PMC10330860

[ref13] Croux C. Haesbroeck G. (2000). Principal component analysis based on robust estimators of the covariance or correlation matrix: influence functions and efficiencies. Biometrika 87, 603–618. doi: 10.1093/biomet/87.3.603

[ref14] Cui J. Zhu D. Su M. Tan D. Zhang X. Jia M.. (2019). The combined use of 1 H and 2D NMR-based metabolomics and chemometrics for non-targeted screening of biomarkers and identification of reconstituted milk. J. Sci. Food Agric. 99, 6455–6461. doi: 10.1002/jsfa.9924, PMID: 31294826

[ref15] Grün J. Papadaki G. Grützkau A. Häupl T. Verginis P. Radbruch A. (2019). “Comparing GCOS-with RMA-HPCDA discovers systematically lowered RMA fold changes” in Technical report. Available at: https://www.researchgate.net/publication/350451210_Comparing_GCOS-with_RMA-HPCDA_discovers_systematically_lowered_RMA_Fold_Changes

[ref16] Güneş A. Mutlu M. Akın İ. Köybaşioğlu F. Güvey A. Karasu M. F.. (2015). The impact of systemic and local administration of ascorbic acid on traumatic perforation of tympanic membrane and myringosclerosis. J. Int. Adv. Otol. 11, 48–52. doi: 10.5152/iao.2015.193, PMID: 26223718

[ref18] Han Y. Liu Y. Tuoya Zhang W. (2024). Protective effect of *Bifidobacterium animalis* subsp. *lactis* BB-69 on mice with inflammatory bowel disease. Chin. J. Microbiol. Immunol. 44, 536–544. doi: 10.3760/cma.j.cn112309-20230530-00146

[ref19] Huang Y. N. Wang J. Y. Lee C. T. Lin C. H. Lai C. C. Wang J. Y. (2012). L-ascorbate attenuates methamphetamine neurotoxicity through enhancing the induction of endogenous heme oxygenase-1. Toxicol. Appl. Pharmacol. 265, 241–252. doi: 10.1016/j.taap.2012.08.036, PMID: 23022510

[ref20] Jin H. Ma T. Chen L. Kwok L. Y. Quan K. Li Y.. (2023). The iLABdb: a web-based integrated lactic acid bacteria database. Sci. Bull. 68, 2527–2530. doi: 10.1016/j.scib.2023.09.016, PMID: 37777465

[ref21] Khailova L. Dvorak K. Arganbright K. M. Halpern M. D. Kinouchi T. Yajima M.. (2009). *Bifidobacterium* improves intestinal integrity in a rat model of necrotizing enterocolitis. Am. J. Physiol. Gastrointest. Liver Physiol. 297, G940–G949. doi: 10.1152/ajpgi.00141.2009, PMID: 20501441 PMC2777452

[ref22] Kim M. Y. Kim E. J. Kim Y. N. Choi C. Lee B. H. (2011). Effects of α-lipoic acid and L-carnosine supplementation on antioxidant activities and lipid profiles in rats. Nutr. Res. Pract. 5, 421–428. doi: 10.4162/nrp.2011.5.5.421, PMID: 22125679 PMC3221827

[ref23] Kim H. Kim J. Kim Y. Jeong Y. Kim J. Paek N.. (2020). Antioxidant and probiotic properties of *lactobacilli* and *Bifidobacterium* of human origins. Biotechnol. Bioprocess Eng. 25, 421–430. doi: 10.1007/s12257-020-0147-x

[ref24] Kurian S. J. Unnikrishnan M. K. Miraj S. S. Bagchi D. Rao M. (2021). Probiotics in prevention and treatment of COVID-19: current perspective and future prospects. Arch. Med. Res. 52, 582–594. doi: 10.1016/j.arcmed.2021.03.002, PMID: 33785208 PMC7972717

[ref25] Li X. Hu D. Tian Y. Song Y. Hou Y. Sun L.. (2020). Protective effects of a novel *Lactobacillus rhamnosus* strain with probiotic characteristics against lipopolysaccharide-induced intestinal inflammation in vitro and in vivo. Food Funct. 11, 5799–5814. doi: 10.1039/D0FO00308E 32568317

[ref26] Li J. King B. K. Janu P. G. Renegar K. B. Kudsk K. A. (1998). Glycyl-L-glutamine-enriched total parenteral nutrition maintains small intestine gut-associated lymphoid tissue and upper respiratory tract immunity. JPEN J. Parenter. Enteral Nutr. 22, 31–36. doi: 10.1177/014860719802200131, PMID: 9437652

[ref27] Li Z. Peng C. Sun Y. Zhang T. Feng C. Zhang W.. (2024). Both viable *Bifidobacterium longum* subsp. *infantis* B8762 and heat-killed cells alleviate the intestinal inflammation of DSS-induced IBD rats. Microbiol. Spectr. 12:e0350923. doi: 10.1128/spectrum.03509-23, PMID: 38647334 PMC11237488

[ref28] Liu L. Hudgins W. R. Shack S. Yin M. Q. Samid D. (1995). Cinnamic acid: a natural product with potential use in cancer intervention. Int. J. Cancer 62, 345–350. doi: 10.1002/ijc.2910620319, PMID: 7628877

[ref29] Nan G. X. Wang L. P. Cui M. J. (2006). Association between cystathionine-beta-synthase gene mutation and ischemic cerebrovascular disease in youths. Chin. J. Clin. Rehab. 10, 170–172. doi: 10.3321/j.issn:1673-8225.2006.10.038

[ref30] Ochiai M. Hayashi T. Morita M. Ina K. Maeda M. Watanabe F.. (2012). Short-term effects of L-citrulline supplementation on arterial stiffness in middle-aged men. Int. J. Cardiol. 155, 257–261. doi: 10.1016/j.ijcard.2010.10.004, PMID: 21067832

[ref31] Ogata H. Goto S. Sato K. Fujibuchi W. Bono H. Kanehisa M. (1999). KEGG: Kyoto Encyclopedia of Genes and Genomes. Nucleic Acids Res. 27, 29–34. doi: 10.1093/nar/27.1.29, PMID: 9847135 PMC148090

[ref32] O’Morain V. L. Ramji D. P. (2020). The potential of probiotics in the prevention and treatment of atherosclerosis. Mol. Nutr. Food Res. 64:e1900797. doi: 10.1002/mnfr.201900797, PMID: 31697015

[ref33] Pereira G. V. Boudaud M. Wolter M. Alexander C. de Sciscio A. Grant E. T.. (2024). Opposing diet, microbiome, and metabolite mechanisms regulate inflammatory bowel disease in a genetically susceptible host. Cell Host Microbe 32, 527–542.e9. doi: 10.1016/j.chom.2024.03.001, PMID: 38513656 PMC11064055

[ref34] Razzaq A. Sadia B. Raza A. Khalid Hameed M. Saleem F. (2019). Metabolomics: a way forward for crop improvement. Metabolites 9:303. doi: 10.3390/metabo9120303, PMID: 31847393 PMC6969922

[ref35] Sun Z. Harris H. M. McCann A. Guo C. Argimón S. Zhang W.. (2015). Expanding the biotechnology potential of *lactobacilli* through comparative genomics of 213 strains and associated genera. Nat. Commun. 6:8322. doi: 10.1038/ncomms9322, PMID: 26415554 PMC4667430

[ref36] Tohge T. Scossa F. Wendenburg R. Frasse P. Balbo I. Watanabe M.. (2020). Exploiting natural variation in tomato to define pathway structure and metabolic regulation of fruit polyphenolics in the *lycopersicum* complex. Mol. Plant 13, 1027–1046. doi: 10.1016/j.molp.2020.04.004, PMID: 32305499

[ref37] Wang C. Han F. Chen X. Zhao A. Wang D. (2022). Time-series based metabolomics reveals the characteristics of the color-related metabolites during the different coloration stages of *Zanthoxylum bungeanum* peel. Food Res. Int. 155:111077. doi: 10.1016/j.foodres.2022.111077, PMID: 35400454

[ref38] Wang H. Lee I. S. Braun C. Enck P. (2016). Effect of probiotics on central nervous system functions in animals and humans: a systematic review. J. Neurogastroenterol. Motil. 22, 589–605. doi: 10.5056/jnm16018, PMID: 27413138 PMC5056568

[ref39] Wang J. Zhao W. Guo S. Sun Y. Yao K. Liu Z.. (2021). Different growth behaviors and metabolomic profiles in yogurts induced by multistrain probiotics of *Lactobacillus casei* Zhang and *Bifidobacterium lactis* V9 under different fermentation temperatures. J. Dairy Sci. 104, 10528–10539. doi: 10.3168/jds.2021-20352, PMID: 34334203

[ref40] Xu J. Xia L. Ni P. (1992). synthesis and cardiovascular activity of Phenylethylamine compounds. J. China Pharm. Univ. 23:6.

[ref41] Zhang L. Li M. Zhan L. Lu X. Liang L. Su B.. (2015). Plasma metabolomic profiling of patients with diabetes-associated cognitive decline. PLoS One 10:e0126952. doi: 10.1371/journal.pone.0126952, PMID: 25974350 PMC4431856

[ref42] Zhao F. Guo Z. Kwok L. Y. Zhao Z. Wang K. Li Y.. (2023). *Bifidobacterium lactis* Probio-M8 improves bone metabolism in patients with postmenopausal osteoporosis, possibly by modulating the gut microbiota. Eur. J. Nutr. 62, 965–976. doi: 10.1007/s00394-022-03042-3, PMID: 36334119

[ref43] Zhou R. Huang Y. Tian C. Yang Y. Zhang Z. He K. (2023). *Coptis chinensis* and berberine ameliorate chronic ulcerative colitis: an integrated microbiome-metabolomics study. Am. J. Chin. Med. 51, 2195–2220. doi: 10.1142/S0192415X23500945, PMID: 37930330

